# Carob (*Ceratonia siliqua*) Flour as Source of Bioactive Compounds: Production, Characterization and Nutraceutical Value

**DOI:** 10.3390/foods13193024

**Published:** 2024-09-24

**Authors:** Iván Benito-Vázquez, Manuel Garrido-Romero, Gema Hontoria-Caballo, Carlos García-García, Marina Díez-Municio, F. Javier Moreno

**Affiliations:** 1 Instituto de Investigación en Ciencias de la Alimentación, CIAL (CSIC-UAM), Nicolás Cabrera 9, 28049 Madrid, Spain; ivan.benito@pharmactive.eu (I.B.-V.); manuel.garrido@pharmactive.eu (M.G.-R.); 2 Pharmactive Biotech Products SLU, Faraday 7, 28049 Madrid, Spain; ghontoria@pharmactive.eu (G.H.-C.); mdiez@pharmactive.eu (M.D.-M.); 3 Centro de Biología Molecular Severo Ochoa, CBM (CSIC-UAM), Nicolás Cabrera, 1, 28049 Madrid, Spain; cgarcia@cbm.csic.es

**Keywords:** bioactive peptides, carob polysaccharides, functional lipids, galactomannan, β-sitosterol

## Abstract

Carob (*Ceratonia siliqua*) seeds are rich in diverse bioactive compounds, including galactomannan, β-sitosterol, unsaturated fatty acids and proteins with bioactive peptides in their sequence. This study delineates the compositional characterization of six carob seed flour types derived from different production processes, providing valuable insights for designing tailored nutraceutical products based on desired bioactive compound profiles. Our analysis indicated that a higher purity of galactomannan resulted in a greater mannose/galactose ratio, which increased the linearity of the galactomannan polymer and could enhance interchain interaction, thereby increasing aggregation capacity. A higher viscosity could potentially increase the capacity of galactomannan to create satiety and lower cholesterol levels. Among the different tested flours, those whose main compound was the endosperm were optimal for containing high galactomannan content, whereas those derived from the germ were ideal for having high concentrations of fatty acids (i.e., oleic and linoleic acids) and β-sitosterol. The presence of these lipids in carob flours could offer cardiovascular and metabolic health benefits, contributing synergistically. Additionally, flours that contain the germ have beneficial peptides included in proteins like glycinin and conglutin with potential anticholesterolemic and antidiabetic properties. This work provides different methods for obtaining carob flours rich in bioactive compounds, offering the nutraceutical industry a framework to select the best option for industrial-scale production.

## 1. Introduction

**Figure 1:**
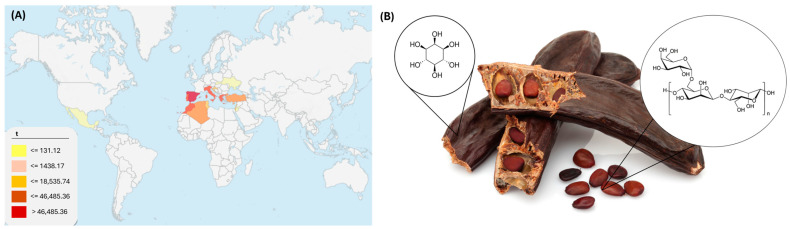
(**A**) Average world carob production in tonnes from 1961 to 2022 (https://www.fao.org/home/es, accessed on 1 August 2024). (**B**) Carob fruit parts, carob pod with inositol, and carob seed with galactomannan.

According to the Food and Agriculture Organization of the United Nations (FAO), historically, Spain has led the production of carob (*Ceratonia siliqua*) in the world with an average production of 126,846 tonnes of carob (Figure 1A). This crop is mainly located in the Mediterranean basin, even though there are other producers like Ukraine or Mexico. Currently, Turkey is the main producer with 25,106 tonnes harvested in 2022, while Spain does not appear in the top 10 producers in that year.

The carob tree is a leguminous plant whose fruit is composed of a pod and seeds (Figure 1B). It is known that carob pods contain various bioactive compounds such as gallic acid and inositol [1,2]. Gallic acid can be found in the seeds, fibers, or pulp and alongside its derivates comprise the majority of phenolic acids of this food, being responsible for glycaemic control and enhanced lipid metabolism, in addition to its proven anticancer effects [3,4]. Likewise, inositol’s biological properties have been thoroughly researched, revealing its roles in insulin regulation and antidiabetic effects. It also exhibits antioxidant and antibacterial activities, enhances female fertility and treats metabolic syndrome. Inositol may also exert beneficial activities as an antidepressant, gastroprotective, hepatoprotective, hypolipidemic and anti-aging agent [5,6].

Additionally, endosperm carob seeds are rich in galactomannan (GM) [7,8], a polysaccharide composed of mannose and galactose whose ratio reported between 3.1 and 3.9 [9] defines its origin and physicochemical properties. It is very effective in contributing to gastrointestinal health and it is used as a thickener, stabilizer of beverages and also as an enhancer of its shelf life [10]. GMs are also used to reduce the fat content of yoghurts [7] and to improve the spreading and texture of cheese [11], apart from being used to control the consistency, mouth feel and stability of the dispersions of food sauces [8]. Finally, their uses in the cosmetic, textile, explosive and pharmaceutical industries are also well studied [12,13,14,15].

Carob flour (CF) and its primary components offer nutritional benefits and could serve as an economical alternative to other ingredients used in fortified food products [16]. This edible fruit is gaining popularity in the creation of innovative functional foods. It is widely used in bakeries due to its potential benefits for human health and the environment [17,18]. The use of CF in producing wheat-free pasta and baked goods was patented in 1935 and the gluten-like properties of this food were also discovered [19]. In addition, it shows a high antioxidant potential, with a demonstrated reduction of myocardial lipid peroxidation and the levels of hydrogen peroxides in kidneys, liver and brain [20]. In addition, an anti-dyslipidemic, anti-obesity and hepatoprotective effect was confirmed in a high-fat-diet-fed rat model [21]. CF also contain lipids like beta-sitosterol [22,23] and proteins that come from its germ [4].

Depending on the production process, CF can have different compounds and, therefore, different nutrients and bioactive properties. If the production process is whole-seed milling, the crude CF obtained contains germ and endosperm bioactive compounds (polysaccharides like galactomannan, lipids like beta-sitosterol and proteins). Instead, seed-dehusking along with endosperm–germ separation offers CF rich in lipids and proteins (carob germ flour) and CF rich in GM (carob endosperm flour) [8,9,24]. From this last flour, further purifications can be carried out by dissolving the GM in hot water and precipitating by using alcohols like isopropanol [7].

The main aim of this work is to compare different industrial production processes of Spanish CFs and their impact on the galactomannan content and properties like mannose/galactose (M/G) ratio and the molecular weight (MW). In addition, a determination of the protein and lipid content along with the study of the protein (SDS-PAGE and proteomics) and lipid profiles (including the quantification of beta-sitosterol) was carried out in order to gain knowledge on the main bioactive molecules and composition characteristics of CFs and, ultimately, on their further nutraceutical applications.

## 2. Methods

### 2.1. CF Production

Different carob seed fractions (Spain) were obtained through dehusking using two methods: (i) boiling carob seed for 1 h and manually dehusking, or (ii) sulfuric acid (Merck, Darmstadt, Germany) 60% treatment in a ratio of 1.6 g seed/mL for 1 h at 60 degrees. Germ and endosperm seed fractions were separated manually from dehusked seeds. Afterwards, whole seed or different seed parts were milled using a lab ball mill model Cryomill (Retsch, Haan, Germany) obtaining CFs (Table 1). In the case of purified GM (Table 1), CF4 was produced through heating a CF1 solution at 10 g/L for 1 h at 80 degrees with magnetic stirring. After that, dissolved GM is separated from the impurities through centrifugation at 4000 rpm for 30 min (Mega Star 1.6/1.6R, VWR, Radnor, PA, USA). The supernatant was mixed with 2 volumes of absolute ethanol for 30 min. Finally, the media was filtered to obtain purified GM, dried overnight at 40 degrees (VL 56 Prime, VWR, PA, USA) and milled. All these samples were analyzed in triplicate.

### 2.2. Galactomannan Quantification

For the determination of the galactomannan concentration per gram of carob flour, a commercial enzymatic kit from Megazyme (Wicklow, Bray, Ireland) was used. A 100 mg amount of carob flour was weighted into a test tube and 5 mL of 80% aqueous ethanol (Merck, Darmstadt, Germany) was added, mixed and incubated at 85–90 °C for 5 min (VL 56 Prime, VWR, Pensilvania, Estados Unidos). Samples were centrifuged at 4000 rpm for 30 min (Mega Star 1.6/1.6R, VWR, Radnor, USA), and the supernatants were discarded. Pellets were resuspended in sodium acetate buffer (Merck, Darmstadt, Germany) (0.1 M, pH 4.5), heated in a boiling water bath with vigorous stirring and cooled to 40 °C. A β-mannanase solution provided in the kit was added and, then, stirred and incubated at 40 °C for 60 min in a water bath (VWR^®^ VWB2, Radnor, PA, USA). Finally, the solution was transferred to a volumetric flask, the volume was adjusted with distilled water and filtered (Whatman^®^ No. 1 filter paper, Merck, Darmstadt, Germany). Detailed protocol and calculations are available at https://www.megazyme.com/, accessed on 1 August 2024.

### 2.3. Carbohydrate Determination by Gas Chromatography

The monomeric composition of the samples was determined with a previous acid hydrolysis of the sample using a strong acid, 2 M trifluoroacetic acid (TFA) (Merck, Darmstadt, Germany). The samples were added into heart-shaped flasks where 2 M TFA was previously added, the oxygen was removed with a nitrogen (N_2_) flux and they were incubated in a stove for 4 h at 110 °C (VL 56 Prime, VWR, PA, USA). Next, the acid was removed thanks to a rotary evaporator (Büchi Labortechnik AG, Flawil, Switzerland) and the internal standard solution (0.5 mg/mL phenyl-β-glucoside; Merck, Darmstadt, Germany) was added, dried and derivatized as shown below before the chromatography analysis.

Trimethyl sialylated oximes (TMSO, Merck, Darmstadt, Germany) of monosaccharides and disaccharides were formed using the method of Ruiz-Matute et al. [25]. First, the mixture of the sample with internal standard solution (0.5 mg/mL phenyl-β-glucoside; Merck, Darmstadt, Germany) was dried in a rotary evaporator (Büchi Labortechnik AG, Flawil, Switzerland). Afterwards, the sugar oximes were formed by adding hydroxylamine chloride (2.5%) in pyridine (Thermo Fisher Scientific, Waltham, MA, USA) and they were agitated and the mixture was heated at 70 °C and 1000 rpm for 30 min. Next, they were sialylated with hexamethyldisilazane (HMDS) (Merck, Darmstadt, Germany) and TFA (Merck, Darmstadt, Germany), and they were heated at 50 °C for 30 min. Finally, the reaction mixture was centrifuged at 10,000 rpm for 5 min at room temperature. The samples were stored at 4 °C until the analysis was performed.

The chromatographic analysis of the TMSO of each sample was performed on Agilent Technologies gas chromatography (GC) (model 7890A) equipped with a flame ionization detector (FID). Separation was carried out in a fused silica capillary column HP-5MS (J&W Scientific, Folsom, CA, USA) and with an automatic injector (Agilent Technologies Inc., Palo Alto, CA, USA). Nitrogen was used as carrier gas at a flow rate of 1 mL/min. Injector and detector temperatures were 280 and 395 °C, respectively. The oven temperature was programmed from 180 to 380 °C at a heating rate of 10 °C/min and held for 20 min. Injections were made in the split mode (1:20). Data acquisition and integration were achieved using Agilent ChemStation 2.3.53 software (Wilmington, DE, USA).

### 2.4. Size-Exclusion High-Performance Liquid Chromatography (HPSEC)

Molecular weight (MW) distribution was estimated following the method described by Muñoz-Almagro et al. using two types of detectors, Evaporative Light Scattering Detector (ELSD) and Refractive Index Detector (RID) [26]. CFs (10 mg/mL) were heated at 80 °C for 1 h in the mobile phase, centrifugated at 13,000 rpm for 5 min and the supernatant was filtered to be separated by HPSEC-ELSD/RID (Agilent Technologies, Boeblingen, Germany) using TSK-Gel guard column (6.0 mm × 400 mm) and two TSK-Gel columns connected in series, G5000 PWXL (7.8 mm × 300 nm, 10 μm) and G2500 PWXL (7.8 mm × 300 nm, 6 μm) (Trosoh Bioscience, Stuttgart, Germany). Sample elution was carried out using 0.01 M NH_4_Ac (Merck, Darmstadt, Germany) min for ELSD and NaNO_3_ (Merck, Darmstadt, Germany) 0.1 M [27] for RID detector as mobile phase at 0.5 mL/min and 30 °C during 50 min. Pullulan standard mix (ReadyCal Kits, Agilent, USA) of MW 1.66 × 10^6^, 2.16 × 10^5^, 2.2 × 10^4^, 991 Da was used as standard.

### 2.5. Sodium Dodecyl Sulfate-Polyacrylamide Gel Electrophoresis (SDS-PAGE)

Samples were resuspended in water to achieve a CF concentration of 10 mg/mL. After that, solutions were centrifugated for 5 min at 13,000 rpm. Then, soluble fractions were mixed well with Laemmli 2X (Bio-Rad, Hercules, CA, USA) containing 2-mercaptoethanol (Thermo Fisher Scientific, MA, USA) at a final volume of 30 μL and heated at 100 °C for 5 min. SDS-PAGE was performed using a MiniProtean Tetra Cell (Bio-Rad, CA, USA) on 12% Mini-PROTEAN^®^ TGX™ Precast Protein Gels (Bio-Rad, CA, USA) with a Tris/glycine/SDS buffer system. The gels were stained with Coomassie blue (Merck, Darmstadt, Germany).

### 2.6. Protein Quantification

Protein concentration was determined by using two different methods:

(i) Bradford was used to quantify soluble protein by dissolving the carob gum in water and centrifugated at 10,000 rpm for 5 min [28]. Then supernatants were mixed with Bradford reagent (Merck, Darmstadt, Germany) and incubated at room temperature for 5 min. The absorbance was measured at 595 nm using an Epoch™ spectrophotometer (Bioteck, Winooski, VT, USA) and bovine serum albumin (Merck, Darmstadt, Germany) was used as a standard to generate a calibration curve.
(ii) DUMAS was used to assess total nitrogen content. This technique involved the combustion of the whole sample in an oxygen-rich, high-temperature chamber. The carbon dioxide (CO_2_), water and produced nitrogen passed through specialized columns that absorb CO_2_ and water. Subsequently, a column equipped with a thermal conductivity detector at the end was used to isolate the nitrogen from any remaining CO_2_ and water, allowing the measurement of the nitrogen content. The equipment used for the protein determination was Trumac 828 Series (Leco, St. Joseph, MO, USA).

### 2.7. Fatty Acid Methyl Ester (FAME) Analysis

The FAME extraction was realized with hexane and samples were derivatized with a specific protocol before the analysis. A mix of 0.5 M sodium methoxide (Merck, Darmstadt, Germany) in methanol (Merck, Darmstadt, Germany) and acetyl chloride (Thermo Fisher Scientific, MA, USA) in methanol (1:10 *v*/*v*) was used as derivatization mix, with C13:0 before methylation being the internal standard (0.5 mg/mL) used for quantification.

For the GC-FID analysis, a GC-26 column 60 m × 250 μm × 0.25 μm (DB-23 model; Agilent, CA, USA) was used with helium as porter gas with a flux of 1 mL/min. The oven temperature was initially 50 °C, then increased at a rate of 25 °C/min to 175 °C, then increased at a rate of 4° C/min to 230 °C and held for 16 min. The samples were injected in split mode (40:1) with a volume of injection of 1 μL.

### 2.8. Soxhlet Fat Extraction

The fat-extraction method was developed using the Soxhlet method, using a Soxtec System HT6 (Tecator AB, Höganäs, Sweden). The samples were weighted into cellulose cartridges and they were placed in continuous extraction vessels where a hot solvent recirculates through them and drags the fat by gravity, being collected in aluminum capsules. The fat percentage was determined by gravimetric calculation of the weights obtained.

### 2.9. β-Sitosterol Determination

The method to quantify β-sitosterol was carried out following the ISO 12228-2:2014 [29] based on the saponification of Soxhlet fat extracted from CFs with potassium hydroxide in ethanol, followed by extraction of the unsaponifiable matter with diethyl ether. Fractionation of the different alcoholic compounds was performed using thin-layer chromatography (TLC) on silica gel to obtain the fraction of interest, which was then converted into trimethylsilyl ether derivatives for subsequent analysis by capillary column gas chromatography (GC) with FID detection. An HP-5 column (5% phenylmethylsiloxane, Agilent) measuring 30 m in length, with an internal diameter of 0.25 mm and a phase thickness of 0.25 microns, was used. The temperature was maintained isothermally at 268 °C for 35 min. Alpha-cholestanol was used as the standard.

### 2.10. Proteomics

#### 2.10.1. Protein Digestion

The protein extracts were resuspended in a volume up to 50 μL of sample buffer and then applied to an SDS-PAGE gel (0.75 mm-thick, 4% stacking and 10% resolving). The unseparated protein bands were visualized by Coomassie staining, excised, cut into cubes (2 × 2 mm) and placed in 0.5 mL microcentrifuge tubes. The gel pieces were detained in acetonitrile:water 1:1, were reduced with 10 mM DTT (Merck, Darmstadt, Germany) for 1 h at 56 °C and alkylated with 10 mM iodoacetamide (Merck, Darmstadt, Germany) for 30 min at room temperature in darkness. After that, they were digested in situ with sequencing grade trypsin (Promega, Madison, WI, USA) as described by Shevchenko et al. with minor modifications [30]. The gel pieces were shrunk by removing all liquid using acetonitrile and the gel pieces were dried in a speedvac concentrator after removing all the acetonitrile. The dried gel pieces were reswollen in 100 mM Tris-HCL pH 8, 10 mM CaCl_2_ with 60 ng/μL trypsin at a protein:enzyme 5:1 (*w*/*w*) ratio. The tubes were kept in ice for 2 h and incubated at 37 °C for 12 h. Digestion was stopped by the addition of 1% TFA. Whole supernatants were dried down and then desalted onto OMIX Pipette tips C18 (Agilent Technologies) until the mass spectrometric analysis.

#### 2.10.2. Reverse Phase–Liquid Chromatography RP-LC-MS/MS Analysis (Dynamic Exclusion Mode)

The desalted protein digest was dried and resuspended in 10 μL of 0.1% formic acid and analyzed via RP-LC-MS/MS in an Easy-nLC II/1200 system coupled to an ion trap LTQ-Orbitrap-Velos-Pro hybrid mass spectrometer (Thermo Scientific, MA, USA). The peptides were concentrated by reverse phase chromatography using a 0.1 mm × 20 mm C18 RP precolumn (Thermo Scientific, MA, USA) and then separated using a 0.075 mm × 250 mm bioZen 2.6 mm Peptide XB-C18 RP column (Phenomenex, Torrance, CA, USA) operating at 0.25 μL/min.

Peptides were eluted using a 90 min dual gradient, whose profile was set as follows: 5–25% solvent B for 68 min, 25–40% solvent B for 22 min, 40–100% solvent B for 2 min and 100% solvent B for 18 min, with solvent A being 0.1% formic acid in water and solvent B 0.1% formic acid, 80% acetonitrile in water. ESI ionization was carried out using a Nano-bore emitter Stainless Stell ID 30 μm (Proxeon, Odense, Copenhagen, Denmark) interface at 2.1 kV spray voltage with S-Lens of 60% and the Orbitrap resolution was set at 30.000 [31].

Peptides were detected in survey scans from 400 to 1600 amu (1 μscan), followed by twenty data-dependent MS/MS scans (Top 20), using an isolation width of 2 u (in mass-to-charge ratio units), normalized collision energy of 35% and dynamic exclusion applied for 60 s periods. Charge-state screening was enabled to reject unassigned and singly charged protonated ions.

#### 2.10.3. Data Processing

Peptide identification from raw data was carried out using PEAKS Studio v11.5 search engine (Bioinformatics Solutions Inc., Waterloo, ON, Canada). A database search was performed against Uniprot-tax-Fabaceae.

The following constraints were used for the searches: tryptic cleavage after Arg and Lys (semi specific), up to two missed cleavage sites, tolerances of 20 ppm for precursor ions and 0.6 Da for MS/MS fragment ions, and allowance of optional Met oxidation and Cys carbamidomethylation. False discovery rates (FDRs) for peptide spectrum matches (PSM) and protein were limited to 0.01. Only those proteins with at least two unique peptides discovered via LC/MS/MS analyses were considered reliably identified.

### 2.11. Statistical Analysis

The results are shown as the mean ± standard deviation (SD) of triplicate observations. Data were analyzed using SigmaPlot 11 statistical software (Systat Software, Richmond, CA, USA). One-way analysis of variance (ANOVA) followed by Tukey’s All Pairwise Comparisons at a 5% confidence level was used to analyze the data (*p* < 0.05).

## 3. Results

### 3.1. Carob Flours

**Figure 2:**
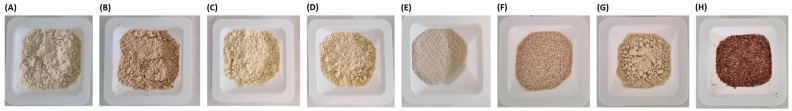
Carob seed flours (CFs). (**A**) Commercial carob flour (C). (**B**) Grounded seed with husk (CF1). (**C**) Grounded seed without husk—water (CF2). (**D**) Grounded seed without husk—sulfuric acid (CF3). (**E**) Purified galactomannan (CF4). (**F**) Grounded endosperm (CF5). (**G**) Grounded germ (CF6). (**H**) Grounded husk (CF7).

Figure 2 displays the tested CFs from the commercial one (left) to the grounded germ (right). Brownish colors were obtained in a CF with husk (CF1, Figure 2B), while CFs were yellowish when the seed germ was present (CF2/3/6, Figure 2C,D,G). On the other hand, whitish CFs were obtained when the seed part was mainly the endosperm or purified galactomannan from it, as CF5 and CF4, respectively (Figure 2E,F). Finally, commercial CF (C, Figure 2A) is a fine seed endosperm flour whose aspect is quite similar to CFs whose main component is the endosperm.

### 3.2. Galactomannan Content and Characterization

**Figure 3:**
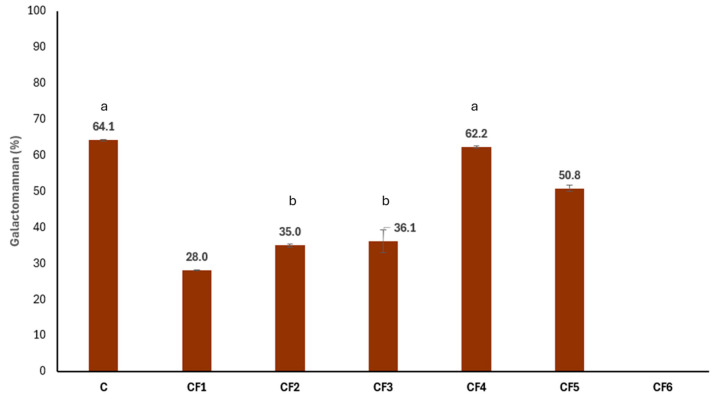
The percentage of galactomannan in the different CFs enzymatically determined. ANOVA test significance, *p* < 0.05. ^a^ Non-significant difference between C and CF4. ^b^ Non-significant difference between CF2 and CF3. C, commercial carob flour. CF1, grounded seed with husk. CF2, grounded seed without husk—water. CF3, grounded seed without husk—sulfuric acid. CF4, purified galactomannan. CF5, grounded endosperm. CF6, grounded germ.

Since GM is the main seed component and the most valuable carob-derived compound for the industry, its quantity per gram was determined in the different CFs (Figure 3). The commercial sample (C) had the highest GM-content CF showing no significant differences with CF4, a purified galactomannan, followed by the CF5 that shared a similar nature with C. This could be due to the milling process which is more efficient in the industrial process than the lab mill. CF1 had the lowest GM-content CF with 28% as the flour is composed of husk, germ and endosperm, with the first two components being GM-free. The two different approaches to dehusking carob seeds (i.e., water versus sulfuric acid) did not affect the GM content as non-significant differences were shown in CF2 and CF3.

The different methods to obtain a variety of CFs in which GM was present as the main bioactive molecule did not significantly affect the mannose/galactose (M/G) ratio as this ranged from 3.0 to 3.5 (Table 2). On the contrary, the GM standard (ST) with a high purity, about 95%, significantly showed the highest M/G ratio compared to the other CFs except for CF4, the purified GM (Table 2). In this sense, it seems that the inclusion of a purification step increases the M/G ratio. The seed-dehusking process did not affect this GM parameter as was previously observed for the GM content [4,32].

SEC analysis indicated that the MWs of GMs were in the range of 10^6^ Da in unimodal distribution (Table 3), which is in line with previous findings reported by other authors for carob [33], guar gums [34] or *Dimorphandra gardneriana* gum [27]. Remarkably, the GM standard also exhibited a higher MW than that reported by the company (i.e., MW about 2 × 10^5^ Da). The most plausible explanation for the observed high MWs of GMs is their tendency to aggregate as previously reported [35]. In fact, using RID/NaNO_3_ did not show significant differences among MW’s samples. However, the ELSD/ammonium acetate method showed CF1 containing the GM with the highest estimated MW and this fact could be due to a higher aggregation compared to ST, CF2, CF4 and CF5.

Finally, CF2 and CF3 were identical in all GM parameters and content, reinforcing that how the two assessed dehusking methods was not important for the GM quality.

### 3.3. Total Protein and Lipid Contents

**Figure 4:**
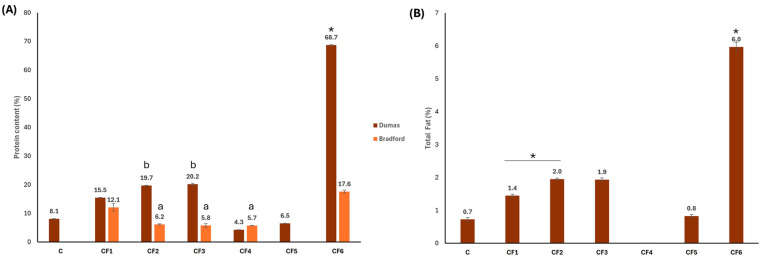
Quantification of (**A**) total and soluble protein content by Dumas (brown) and Bradford (orange) methods, respectively; and (**B**) total fat content by Soxhlet method. ANOVA test significance, *p* < 0.05. * Significant differences (*p* < 0.05). C, commercial carob flour. CF1, grounded seed with husk. CF2, grounded Seed without husk—water. CF3, grounded seed without husk—sulfuric acid. CF4, purified galactomannan. CF5, grounded endosperm. CF6, grounded germ. ^a^ Non-significant difference between CF2, CF3 and CF4. ^b^ Non-significant difference between CF2 and CF3.

CF5 and C contained a very low or negligible protein content (Figure 4A). This finding was expected by considering their origin, the seed endosperm which is protein-free. On the other hand, despite being a purified galactomannan, CF4 was shown to have a residual soluble protein content present in the other CFs due to the production process in which the precipitation step recovered not only GM but also proteins. CF2 and CF3 were identical in soluble and total protein content, highlighting again the respectfulness of both dehusking methods, the aqueous and acidic, in terms of protein content. CF6 had the highest protein content due to its germ-seed origin that is rich in protein, while the other CFs contained a lower protein content since the major component was the galactomannan-rich endosperm and not protein and lipids like in the germ. Furthermore, the notable difference observed mainly in CF6 between the Dumas and Bradford methods for protein determination was expected because the former determined both soluble and insoluble proteins present in the grounded seed germ. In contrast, CF4 Bradford and Dumas values were identical due to the production process mentioned before.

Regarding total fat content, CF4 had no fats (Figure 4B), revealing that this production process is suitable for obtaining CFs that are rich in galactomannan, have low protein content and are free fat. As in previous determinations, CF2 and CF3 fat content were identical and the dehusking process did not affect the total free fat content. The maximum lipid content was obtained in CF6 (i.e., 6%), with the grounded germ being mainly rich in protein and lipids, followed by CF2 and CF3 with a lipid content of about 2%.

### 3.4. Protein and Lipid Profiles

**Figure 5:**
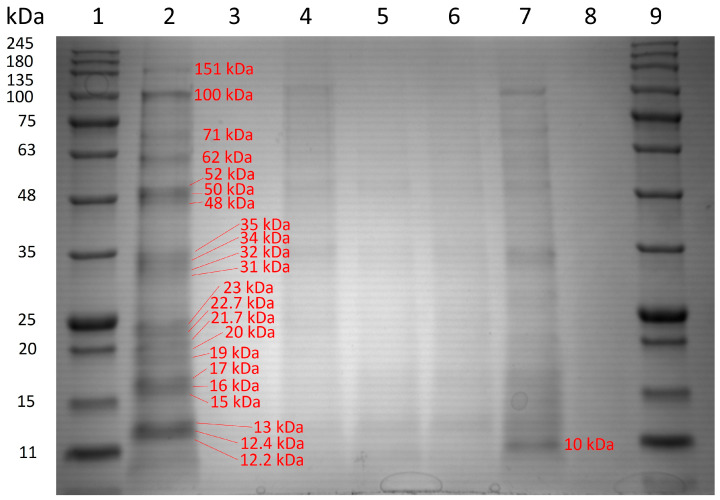
Denaturing SDS-PAGE 12% polyacrylamide gel. Lane 1, protein marker. Lane 2, grounded carob germ (CF6). Lane 3, grounded carob endosperm (CF5). Lane 4, purified galactomannan from whole seed without husk (CF4). Lane 5, grounded carob seed without husk—acid treatment (CF3). Lane 6, grounded carob seed without husk—water treatment (CG2). Lane 7, grounded carob seed with husk (CF1). Lane 8, commercial carob gum (C). Lane 9, protein marker.

A similar qualitative pattern of soluble proteins was observed by SDS-PAGE for all produced CFs (Figure 5). However, the sample CF6, which is based on grounded germ, showed the most intense bands, which is in good agreement with the total protein content estimated by the Dumas method (Figure 4A). In line with previous works [36], the carob germ has four major bands detected under denaturing conditions with β-mercaptoethanol and extracted with an organic solvent and heat. Those bands migrated at 150, 97, 50 and 28 kDa in 12% electrophoresis gel. The last two bands could correspond to 11S globulins present in other Leguminosae species according to the theoretical MW and previous findings. This tentative identification was further corroborated by proteomics analysis in the CF6 sample (Table 4).

Proteomics analysis identified 1243 proteins including conglutins α/β and glycinins G1/G3 (Table 4). These proteins have been reported in the bibliography as potential sources of bioactive peptides formed after digestion. Since these proteins were present in CF6, they were supposed to be also present in CF1, CF2 and CF3 as they contained the seed germ. Therefore, all these CFs would have the bioactive properties attributable to these proteins and derived peptides.

**Figure 6:**
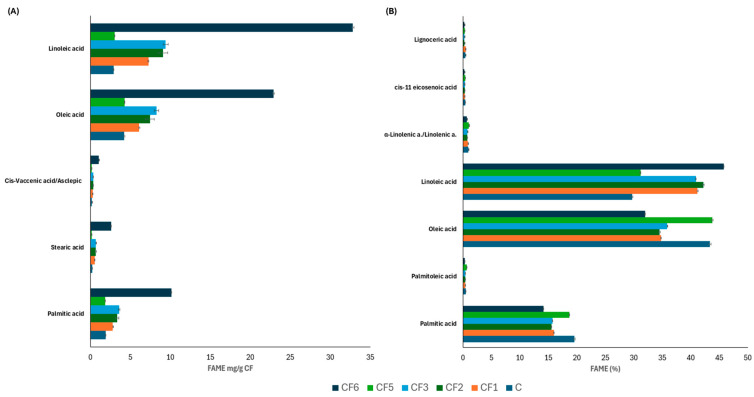
Main fatty acid methyl ester (FAME) content in milligrams per 100 g of CF (at the bottom). (**A**) in milligrams per gram of carob flour and (**B**) percentage. C, commercial carob flour. CF1, grounded seed with husk. CF2, grounded seed without husk—water. CF3, grounded seed without husk—sulfuric acid. CF4, purified galactomannan. CF5, grounded endosperm. CF6, grounded germ.

The lipid profile was determined in all lipid-content CFs (Supplementary Material Tables S1 and S2). Thus, 16 fatty acids (FAs) were detected in CF6 (71.6 mg/g) followed by 15 in CF1 (17.6 mg/g), CF2 (21.54 mg/g) and CF3 (23.04 mg/g). C (9.81 mg/g) and CF5 (9.82 mg/g) had the same lipid profile and the same total fatty acid in milligrams per gram as they came from the seed endosperm. In CF1, CF2, CF3 and CF6, 37.5% of detected FAs were unsaturated and 33% of them were polyunsaturated (palmitoleic, cis-vaccenic, linoleic, oleic, alpha-linolenic and cis-11 eicosenoic acid). Linoleic, oleic and palmitic acids constituted nearly 100% of the fatty acids detected in the samples (Figure 6B), reaching respective values of 32.81, 22.91 and 10.15 mg/g in CF6, whereas in CF2 and CF3 they had a similar content of 9, 8.5 and 3.5 mg/g. Finally, CF1 had slightly lower values of these FAs as compared to CF2 and CF3 (Figure 6A).

Moreover, as could be expected due to its highest content in total fat, CF6 contained the largest quantity of β-sitosterol (i.e., 98.0 mg/100 g CF), followed distantly by CF1 (41.5 mg/100 g CF), CF2/CF3 (26.1 mg/100 g CF), C (22.9 mg/100 g CF) and CF5 (16.9 mg/100 g CF).

## 4. Discussion

Carob seeds are known to have a great variety of bioactive compounds of different natures. Polysaccharides like galactomannan, sterols like β-sitosterol, fatty acids like oleic, palmitic or linoleic acids, as well as some proteins homologous to those present in other leguminous species that produce bioactive peptides during digestion are shown in Table 4 [7,8,22]. In this work, we carried out the production of CFs using several processes to obtain a great variety of CFs with different components and concentrations in order to offer possibilities for the manufacturing in nutraceutical industry.

Considering the quantity and quality of GM, we observed that a higher purity results in a greater M/G ratio, which increases the linearity of the GM polymer and could enhance interchain interaction, thereby increasing aggregation capacity as we can see in Table 3 [45]. However, despite being more purified [7,8], CF4 did not contain a GM with a higher MW than the other produced CFs. However, in terms of GM quality, all CFs produced in this study are similar to and slightly better than the commercial product (C) used as control. The M/G ratio and MW are critical factors as they determine the physicochemical properties of galactomannan [46]; thus, an increasing GM viscosity is associated with a higher satiating mass in the stomach when ingested. This property is utilized by many commercial products in the field of functional foods for weight control and LDL reduction, improving the lipid profile since this indigestible polysaccharide mass can bind to cholesterol and triglycerides (TG), preventing their absorption in the gut [47,48,49]. However, the produced flours differed in their GM concentration, so if GM is the target compound in the final manufactured product, processes like those yielding CF4 and CF5 would be the most optimal. In fact, there are in vivo studies for nutraceutical products standardized in GM where TG and LDL levels were decreased after the treatment in mice with induced metabolic syndrome [50,51]. However, if the presence of other lipid and protein compounds is desired, CF4 and CF5 are not the best options, as they have the lowest values for these types of compounds (Figure 4). In this case, production processes used to obtain CF1, CF2 or CF3 would be more optimal for maintaining GM and including lipid-derived compounds, such as oleic and linoleic acids which were the most abundant in the lipid fraction [22,23]. Furthermore, if a high concentration of these fatty acids is sought, the optimal production process would be the one that yields CF6, a flour from the germ of the carob seed, which does not contain GM (Figure 3). Oleic and linoleic acids offer numerous benefits in the field of nutraceuticals due to their essential roles in human health. Oleic acid, a monounsaturated omega-9 fatty acid, is known to reduce the risk of cardiovascular diseases by lowering LDL cholesterol levels while maintaining or increasing HDL cholesterol levels, and it also has anti-inflammatory and antioxidant properties that reduce chronic inflammation and oxidative stress [52,53,54]. Additionally, it has been linked to improved insulin sensitivity and reduced risk of type 2 diabetes [55] and supports the immune system by enhancing immune function and modulating inflammatory responses [56]. Furthermore, linoleic acid contributes to heart health by lowering LDL cholesterol levels and reducing the risk of coronary heart disease when consumed as part of a balanced diet [57]. Together, these fatty acids contribute to overall health and wellness, making them valuable components in nutraceutical products aimed at enhancing quality of life and preventing chronic diseases.

β-sitosterol follows a similar pattern to that of the unsaturated fatty acids, changing slightly for CF1, CF2 and CF3; however, CF6 would be the preferable choice in search of a product with a high β-sitosterol concentration. This compound offers numerous benefits in the field of nutraceuticals similar to oleic and linoleic acids. It is well known for its ability to improve cardiovascular health by lowering cholesterol and TG levels in in vivo models, thereby reducing the risk of heart disease [58,59]. In addition, it possesses anti-inflammatory properties, which can help alleviate chronic inflammation linked to various diseases, including arthritis [60] and metabolic syndrome [61].

Finally, regarding protein profile, the identification of specific peptides was difficult due to the lack of a reference genome for carob. The proteins identified by proteomics were based on a higher-order database, *Fabaceae*, using a strategy that detects all present proteins. Therefore, although SDS-PAGE protein profiles described in other studies allow the tentative identification of the flour’s origin, they did not permit the detection of specific peptides or proteins of interest beyond fragments of the 11S globulin based on the literature (Figure 5). However, the proteomic analysis of CF6 allowed the identification of peptides from proteins as glycinin [40,41,42,43,44] and conglutin [37,62,63,64], which are of interest in supplementation for their anticholesterolemic and antidiabetic properties, as shown in Table 4. Again, this flour would offer a higher concentration of these peptides in the product due to its high content in protein (Figure 4A), although their presence at lower concentrations could also be obtained in flours such as CF1, CF2 and CF3.

To conclude, this work examines a comprehensive array of bioactive compounds found in carob flours produced through various methods, determining their concentration and emphasizing the potential synergistic effects that they may have in the final product. These bioactive compounds are a collection of various biomolecules such as galactomannan, with a high molecular weight and a high M/G ratio, whose bioactive properties focus on reducing TG and LDL levels. Additionally, there are bioactive peptides derived from major proteins in legumes, which have antidiabetic and cholesterol-lowering effects. Similarly, beta-sitosterol, due to its structural similarity to cholesterol, also helps to reduce blood cholesterol levels. Moreover, the presence of fatty acids such as oleic, palmitic and linoleic acids further contributes to the antidiabetic effects of the bioactive peptides and the cholesterol-lowering effect of the galactomannan, bioactive peptides and beta-sitosterol.

Thus, we provide different methods for obtaining carob flours rich in bioactive compounds, offering the nutraceutical industry a framework to select the best option for industrial-scale production. As an example of an appealing application in nutraceuticals, this selection could be tailored to the final product’s goals in weight management and lipid profile improvement, leveraging the synergistic effects of the different bioactive molecules contributing to those beneficial effects.

## 5. Conclusion

Carob flours have great potential as a nutraceutical ingredient, and they can be enriched in different bioactive compounds depending on the starting material and production process. They contain an endosperm rich in galactomannan, which has a satiating effect and reduces cholesterol and LDL levels. Additionally, the germ is rich in lipids, which have a similar effect, and proteins, which encompass peptides with antidiabetic potential and lipid profile-improving properties. These bioactive compounds could have a combined effect in reducing LDL and cholesterol levels, as well as improving the cardiometabolic health of individuals who consume them. Therefore, our findings warrant further investigation to gain insights into the in vivo effects of carob flours enriched in a variety of bioactive compounds.

## Figures and Tables

**Table 1:** Different carob flours (CFs) obtained from carob seeds through various techniques.

Sample	Code
Commercial carob flour	C
Grounded carob seed with husk	CF1
Grounded carob seed without husk removed with water treatment	CF2
Grounded seed without husk removed with acid treatment	CF3
Purified carob seed galactomannan	CF4
Grounded carob seed endosperm	CF5
Grounded carob seed germ	CF6

**Table 2:** Galactomannan mannose/galactose ratio in the different carob flours measured by GC-FID.

Carob Flour Sample	Mannose/Galactose Ratio	Standard Deviation
Galactomannan Standard ^a^	3.9	0.03
C ^a^	3.0	0.07
CF1 ^b^	3.4	0.16
CF2 ^a^	3.4	0.29
CF3 ^a^	3.3	0.05
CF4	3.5	0.03
CF5 ^a^	3.4	0.07

^a^ Significant difference between standard and C/CF2/CF3/CF5 (*p* < 0.050). ^b^ Slight difference between ST and CF1 (*p* = 0.075). C, commercial carob flour. CF1, grounded seed with husk. CF2, grounded seed without husk—water. CF3, grounded seed without husk—sulfuric acid. CF4, purified galactomannan. CF5, grounded endosperm. CF6, grounded germ.

**Table 3:** Molecular weight determination using SEC in the different CFs by two methods.

Sample	HPLC Detector/Mobile Phase	Molecular Weight (Da)	SD
Standard ^a,b,c^	ELSD/Ammonium acetate 0.01 M	1.99 × 10^6^	2.01 × 10^5^
C ^b^		3.38 × 10^6^	2.11 × 10^5^
CF1 ^a^		3.57 × 10^6^	3.87 × 10^5^
CF2 ^a,b^		2.51 × 10^6^	3.31 × 10^4^
CF3 ^c^		2.99 × 10^6^	2.27 × 10^5^
CF4 ^a,b^		2.52 × 10^6^	4.07 × 10^4^
CF5 ^a,b^		2.29 × 10^6^	4.79 × 10^4^
Standard	RID/NaNO_3_ 0.1 M	2.17 × 10^6^	1.89 × 10^5^
C		2.96 × 10^6^	4.02 × 10^5^
CF1		3.28 × 10^6^	8.50 × 10^5^
CF2		3.61 × 10^6^	3.84 × 10^5^
CF3		3.44 × 10^6^	1.48 × 10^5^
CF4		3.33 × 10^6^	3.22 × 10^5^
CF5		2.94 × 10^6^	1.26 × 10^5^

ANOVA test significance, *p* < 0.05. ^a^ Significance difference CF1 vs. ST/CF2/CF4/CF5. ^b^ Significance difference C vs. ST/CF2/CF4/CF5. ^c^ Significance difference CF3 vs. ST. C, commercial carob flour. CF1, grounded seed with husk. CF2, grounded Seed without husk—Water. CF3, grounded seed without husk—sulfuric acid. CF4, purified galactomannan. CF5, grounded endosperm. CF6, grounded germ.

**Table 4:** LC-MS2-based proteomics identification of selected proteins present in grounded carob seed germ (CF6).

Uniprot Entry	Identification	Theoretical MW (kDa)	Potential Bioactive Properties
F5B8V7|CONA2_LUPAN	Conglutin alpha 2	2A subunit (51.4)/2B subunit (19.1)	Satiety and weight loss [37] LDL and VLDL reduction [38]
F5B8W5|CONB7_LUPAN	Conglutin beta 7	68.3	Satiety and weight loss [37] Antidiabetic effect and total serum cholesterol reduction [39] LDL and VLDL reduction [38]
P11828|GLYG3_SOYBN	Glycinin G3	A1b subunit (31.5)/B2 subunit (19.9)	Potential antidiabetic effect through α-amylase, α-glucosidases inhibition and dipeptidyl peptidase IV inhibition [40] Glucose metabolism modulation [41] Cholesterol metabolism modulation [42] HDL increase and triglycerides reduction [43,44]
A0A445FHF7|A0A445FHF7_GLYSO	Glycinin G3	52.2	
P04776|GLYG1_SOYBN	Glycinin G1	A1a subunit (32.7)/Bx subunit (19.9)	
A0A834TBD1|A0A834TBD1_9FABA	Glycinin G3	58.7	
A0A6P4DH65|A0A6P4DH65_ARADU	11S globulin	46.9	N/A

## Data Availability

The data presented in this study are available on request from the corresponding author (F.J.M.).

## Supplementary Materials

The following supporting information can be downloaded at: https://www.mdpi.com/article/10.3390/foods13193024/s1, Table S1: Fame fatty acid methyl esters (FAME) in milligrams per gram of carob flour; Table S2: Fame fatty acid methyl esters (FAME) in percentage.
