# Supplementary material for: Carob (Ceratonia siliqua) Flour as Source of Bioactive Compounds: Production, Characterization and Nutraceutical Value
Source: Foods. 2024 Sep 24;13(19):3024. doi: 10.3390/foods13193024 (PMC11475722; doi:10.3390/foods13193024)
Supplement: Supplementary file 1 [file foods-13-03024-s001.zip › foods-3195810-supplementary.pdf]

Table S1. Fame fatty acid methyl esters (FAME) in milligrams per gram of carob flour.

	mg FAME/g CF											
**MUESTRA**	**C**	**SD**	**CF1**	**SD**	**CF2**	**SD**	**CF3**	**SD**	**CF5**	**SD**	**CF6**	**SD**
Myristic acid	0.02	0.00	0.02	0.00	0.02	0.00	0.03	0.00	0.00	0.00	0.07	0.00
Pentadecylic acid	0.03	0.00	0.02	0.00	0.02	0.00	0.03	0.00	0.02	0.00	0.06	0.00
Palmitic acid	1.92	0.03	2.82	0.02	3.34	0.24	3.61	0.11	1.84	0.00	10.15	0.03
Palmitoleic acid	0.05	0.00	0.07	0.00	0.09	0.01	0.09	0.00	0.06	0.00	0.22	0.00
Margaric acid	0.00	0.00	0.03	0.00	0.03	0.00	0.03	0.00	0.00	0.00	0.11	0.00
Stearic acid	0.23	0.02	0.55	0.01	0.70	0.05	0.71	0.02	0.18	0.00	2.58	0.01
Cis-Vaccenic acid/Asclepic	0.20	0.00	0.28	0.00	0.34	0.02	0.36	0.01	0.18	0.00	1.07	0.01
Oleic acid	4.25	0.08	6.12	0.05	7.44	0.54	8.26	0.26	4.30	0.02	22.91	0.10
Linoleic acid	2.91	0.05	7.25	0.07	9.09	0.61	9.42	0.29	3.06	0.02	32.81	0.12
α-Linolenic a./Linolenic a.	0.10	0.00	0.15	0.00	0.17	0.01	0.18	0.01	0.10	0.00	0.50	0.00
Arachidic acid	0.00	0.00	0.07	0.00	0.08	0.01	0.08	0.00	0.00	0.00	0.33	0.00
cis-11 eicosenoic acid	0.04	0.00	0.05	0.00	0.06	0.00	0.07	0.00	0.04	0.00	0.20	0.01
Heneicosanoic acid	0.00	0.00	0.00	0.00	0.00	0.00	0.00	0.00	0.00	0.00	0.06	0.00
Behenic acid	0.00	0.00	0.04	0.00	0.05	0.00	0.05	0.00	0.00	0.00	0.19	0.00
Tricosanoic acid	0.03	0.00	0.04	0.00	0.05	0.00	0.05	0.00	0.02	0.00	0.19	0.00
Lignoceric acid	0.04	0.00	0.08	0.00	0.06	0.00	0.06	0.00	0.03	0.00	0.19	0.00
**TOTAL**	9.81		17.60		21.54		23.04		9.82		71.63	

C, commercial carob flour. CF1, grounded seed with husk. CF2, grounded Seed w/o husk – Water. CF3, grounded seed w/o husk – sulfuric acid. CF5, grounded endosperm. CF6, grounded germ.

Table S2. Fame fatty acid methyl esters (FAME) in percentage.

	Percentage FAME (%)											
**MUESTRA**	**C**	**SD**	**CF1**	**SD**	**CF2**	**SD**	**CF3**	**SD**	**CF5**	**SD**	**CF6**	**SD**
Myristic acid	0.19	0.01	0.13	0.00	0.11	0.00	0.11	0.00	0.00	0.00	0.09	0.00
Pentadecylic acid	0.28	0.01	0.13	0.01	0.11	0.01	0.11	0.00	0.19	0.01	0.08	0.00
Palmitic acid	19.57	0.14	16.01	0.05	15.53	0.04	15.68	0.01	18.69	0.04	14.16	0.02
Palmitoleic acid	0.50	0.01	0.41	0.01	0.40	0.00	0.41	0.00	0.64	0.00	0.31	0.00
Margaric acid	0.00	0.00	0.17	0.00	0.14	0.00	0.14	0.00	0.00	0.00	0.15	0.00
Stearic acid	2.35	0.23	3.11	0.03	3.23	0.01	3.06	0.01	1.81	0.01	3.61	0.00
Cis-Vaccenic acid/Asclepic	2.03	0.02	1.58	0.01	1.59	0.01	1.57	0.00	1.79	0.02	1.49	0.00
Oleic acid	43.35	0.24	34.75	0.02	34.53	0.14	35.85	0.02	43.76	0.13	31.98	0.01
Linoleic acid	29.67	0.15	41.19	0.06	42.21	0.14	40.89	0.01	31.17	0.10	45.80	0.02
α-Linolenic a./Linolenic a.	0.97	0.01	0.88	0.01	0.77	0.01	0.79	0.00	1.04	0.00	0.69	0.00
Arachidic acid	0.00	0.00	0.38	0.01	0.39	0.01	0.37	0.01	0.00	0.00	0.47	0.00
cis-11 eicosenoic acid	0.38	0.00	0.30	0.00	0.29	0.00	0.31	0.00	0.38	0.01	0.28	0.01
Heneicosanoic acid	0.00	0.00	0.00	0.00	0.00	0.00	0.00	0.00	0.00	0.00	0.09	0.00
Behenic acid	0.00	0.00	0.25	0.02	0.22	0.01	0.21	0.01	0.00	0.00	0.27	0.00
Tricosanoic acid	0.00	0.01	0.24	0.02	0.23	0.01	0.23	0.01	0.19	0.01	0.27	0.01
Lignoceric acid	0.00	0.01	0.47	0.03	0.26	0.00	0.26	0.01	0.34	0.02	0.27	0.00

C, commercial carob flour. CF1, grounded seed with husk. CF2, grounded seed w/o husk – Water. CF3, grounded seed w/o husk – sulfuric acid. CF5, grounded endosperm. CF6, grounded germ.
